# Facial icons as indexes of emotions and intentions

**DOI:** 10.3389/fpsyg.2024.1356237

**Published:** 2024-05-14

**Authors:** Amel Achour-Benallegue, Jérôme Pelletier, Gwenaël Kaminski, Hideaki Kawabata

**Affiliations:** ^1^Cognition, Environment and Communication Research Team, Human Augmentation Research Center, National Institute of Advanced Industrial Science and Technology, Kashiwa, Japan; ^2^Institut Jean Nicod, Département d'études cognitives, ENS, EHESS, CNRS, PSL University, Paris, France; ^3^Department of Philosophy, University of Western Brittany, Brest, France; ^4^Cognition, Langues, Langage, Ergonomie, Université de Toulouse, Toulouse, France; ^5^Institut Universitaire de France, Paris, France; ^6^Department of Psychology, Faculty of Letters, Keio University, Tokyo, Japan

**Keywords:** embodied emotion, emotional contagion, facial mimicry, theory of mind, face representations, art agency, art perception

## Abstract

Various objects and artifacts incorporate representations of faces, encompassing artworks like portraits, as well as ethnographic or industrial artifacts such as masks or humanoid robots. These representations exhibit diverse degrees of human-likeness, serving different functions and objectives. Despite these variations, they share common features, particularly facial attributes that serve as building blocks for facial expressions—an effective means of communicating emotions. To provide a unified conceptualization for this broad spectrum of face representations, we propose the term “*facial icons”* drawing upon Peirce’s semiotic concepts. Additionally, based on these semiotic principles, we posit that facial icons function as indexes of emotions and intentions, and introduce a significant anthropological theory aligning with our proposition. Subsequently, we support our assertions by examining processes related to face and facial expression perception, as well as sensorimotor simulation processes involved in discerning others’ mental states, including emotions. Our argumentation integrates cognitive and experimental evidence, reinforcing the pivotal role of facial icons in conveying mental states.

## 1 Introduction

The term *facial icon* is usually associated with digital face illustrations such as avatars (Leone, 2018). However, in this paper we use the word *facial icon* to define a broader category of face representations. A *facial icon* could be a face in artistic or ethnographic sculptures, comic strips, manga, or characters in animated films. It can also be a mask, a bust, a portrait or any face-like representation. Furthermore, a facial icon may take the form of a component integrated into a machine, such as a robot or an automaton. *The facial icon* category encompasses artifacts^1^ that portray faces as conspicuous components of a body. They constitute either complete entities or components of anthropomorphic artifacts^2^ which are visual objects of the human appearance representation. Anthropomorphic artifacts are characterized by abundant and nearly ubiquitous presence in the history of figuration (Achour-Benallegue et al., 2016).^3^

As numerous as they are diverse, *facial icons* cover a broad spectrum of human-likeness, with deformations at times perceived as an enhancement of expressiveness (Lacoue-Labarthe et al., 2008) and esthetics (Ramachandran and Hirstein, 1999). They portray various expressions that can be characterized as intense and appealing. As articulated by De Smedt and Jucker (2018) “Successful visual art appeals to us because it exaggerates or appropriates features that human perception is attuned to (e.g., color contrasts, contours).” This prompts consideration that the appeal of facial icons lies not in the essence of the representation itself but in its relevance to humans. If an artifact depicting the human form, particularly facial expressions, appears highly expressive to us, it is likely due to its capacity to convey not only a narrative through representation but also the narrative of our own interaction with each other. Some philosophers posit that the cultural success of facial icons such as masks and portraits can be attributed to “the evolutionary salience of face detection for humans – highly social animals that put a premium on individual recognition.” (De Smedt and De Cruz, 2010). The archeologist Matsumoto argued that “anthropomorphic things are generally appealing to us because they are at the nexus of two kinds of cognitive domains: social and technical […] social cognition consists of a number of skills including […] recognition of social relationships and communication signals, such as facial expressions, and understanding other’s intentions” (Matsumoto, 2021, p. 64–65). In alignment with these scholars, we assert that *facial icons* capture the attention of individuals across diverse human societies because they reflect a recognizable configuration and a pertinent sign universally. Indeed, the portrayal of facial expressions represents configurations that are highly captivating and would serve as a profoundly relevant semiotic medium, given that facial expressions constitute a highly significant non-verbal language for communicating emotions (Awasthi and Mandal, 2015; Gallese, 2022). This is likely the factor that makes them a universally shared iconography, as evidenced in the representation across various cultures. De Smedt and De Cruz (2011) proposed that what is common to art behavior in disparate cultures may be elucidated by stable features of human cognition. We argue that the representation and reception of facial icons across cultures benefit from the processing of faces and facial expressions.

The expressiveness of artifacts and their significance in society is typically examined within the realms of archeology, anthropology, or art. However, the examination of facial expressions and the impressions and reactions they elicit in perceivers is more closely associated with psychological inquiries. In this article, we will introduce a transitional step, connecting an anthropological framework to a cognitive psychology perspective through the lens of semiotics. Earlier studies endeavored to establish connections between cognitive processing and the cultural evolution of portraits (Morin, 2013), or sought to elucidate the saliency of representations of the human face, such as in masks, portraits, and busts, by invoking cognitive processes (De Smedt and De Cruz, 2010, 2011; De Smedt and Jucker, 2018). Our contribution may serve as complementary building blocks that could offer insights into the robust and sustained portrayal of facial expressions from a cognitive perspective.

## 2 Facial icon: a semiotic definition

To the best of our knowledge, there has not been a specific proposal for a precise definition to characterize faces within a cross-cultural array of anthropomorphic artifacts. The category of facial icons encompasses both artistic objects (artworks) and ethnographic or industrially manufactured objects, whose functionality may not inherently align with artistic purposes. Our definition of this category seeks to encompass, under the same classification of objectual entities, works of art, cinematographic characters, ethnographic objects, and objects of industrial manufacture. This approach facilitates the manipulation of diverse entities based on a shared common denominator.

**Figure 1:**
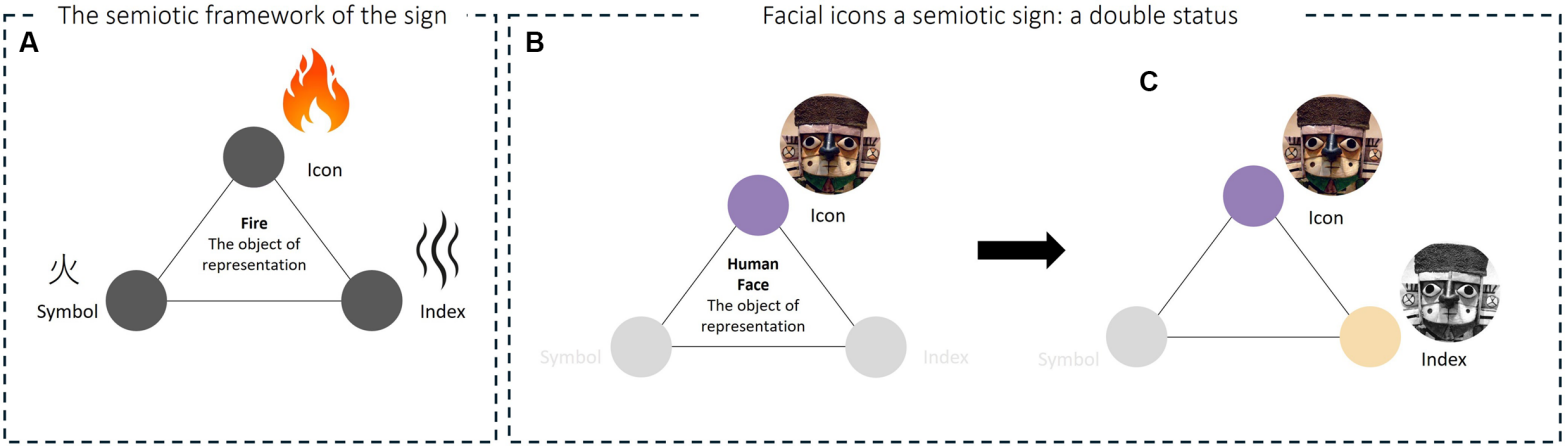
Representation of facial icons in the realm of semiotics. **(A)** An Example of Sign Statuses Posited by Peirce. Here, the object “fire” can be represented by (i) Peirce’s semiotics as a flame (icon), (ii) smoke (index), or (iii) a Chinese ideogram that designates the word “fire” (symbol). **(B)** Iconic Presentation of The Human Face. The face representation within the anthropomorphic artifact, exemplified here by the face of a Huari sculpture, resides within the icon category of Peirce’s semiotic framework. This icon functions as a sign, signifying a relationship of resemblance with the human face. **(C)** Indexical Presentation of Facial Expression. When the facial icon is related to a perceiver, it assumes the position of the Index in Peirce’ semiotic network (indicated by the orange circle). While it continues to function as an icon (depicted by the purple circle) in the representation of the human face, it concurrently transforms into an index conveying (human) emotions and intentions. The Huari sculpture picture used in this figure belongs to public domain, available at: https://commons.wikimedia.org/wiki/File:Figurine_of_dignitary,_Wari_-_Peru.jpg.

The terminology employed for facial icons, as we propose, draws inspiration from Peirce’s semiotics. Within Peirce’s semiotic theory of signs (Peirce, 2006), any object is situated within a network of semiotic relations, with the sign serving as the fundamental building block. A sign can be categorized as an icon, an index, or a symbol (refer to Figure 1A). (a) An icon is a sign that maintains a relationship of resemblance with the object. (b) An *index* is a sign that maintains a relationship of causal connection with the object, indicating the cause of its existence. (c) A *symbol* is a sign which maintains a conventional relation with the object and is imbued with abstract significance. We have chosen to characterize our category of anthropomorphic artifacts by employing the concept of “icon,” not solely based on the use of “facial icon” reference in digital face illustrations but because the concept of icon, in its extension, encompasses artifacts of diverse natures. Moreover, it provides the opportunity to examine these artifacts within the same “signifying universe” (Esquenazi, 1997). Consequently, we define this class of anthropomorphic artifacts as an iconic presentation of the human face (see Figure 1B). This implies that we regard the depiction of the face in anthropomorphic artifacts as an icon in Peirce’s sense, wherein it represents the “human face” as a physical appearance. In this context, we refer to a material resemblance, despite its varying degrees of human-likeness. In succinct terms, a facial icon is, by definition, a poietic visual sign^4^ that replicates, to varying extents of human-likeness, the appearance of the human face.

## 3 From the icon to the index

*A face is a center of human expression, the transparent envelope of the attitudes and desires of others, the place of manifestation, the barely material support for a multitude of intentions* (Merleau-Ponty and Wild, 1963).

Facial icons, regardless of their degree of human-likeness, inherently possess qualities that distinguish them almost ontologically from other representation categories. The inherent human attributes and their underlying expressiveness confer a spiritual status upon these anthropomorphic artifacts.^5^ Moreover, facial expressions endow them with a communication profile capable of conveying emotions and intentions.

The human face, as per definition, serves as the subject of the iconic relationship represented by the facial icon. However, the face, as a component of the body, is far from neutral in expressing an individual’s mental states. The human face possesses the capacity to convey information about certain aspects of a person’s mental state solely based on its structural configuration (Frijda, 2008). Indeed, during interpersonal interactions, facial expressions communicate an individual’s feelings or intended messages. Among non-verbal forms of communication, facial expressions prove to be the most effective means of conveying emotions (Awasthi and Mandal, 2015; Tarnowski et al., 2017). Moreover, they are the focal point of individuals’ intentions behind their expressions (Rasgado-Toledo et al., 2021). For instance, a smile can signify a friendly disposition and, more broadly, express a motivation for affiliation, indicating an intention to foster or maintain social bonds (Martin et al., 2017). Therefore, the face serves as a highly effective mediator of individuals’ intentions and emotions.

As the configuration of facial features remains distinguishable in artifacts despite variations in their degree of human-likeness, we propose the extension of the capacity to communicate emotions and intentions through facial expressions to facial icons. This implies that facial icons, through their portrayed expressions, can serve as an effective means of conveying emotions and intentions, considered as mental states. However, it is essential to note that facial icons lack the intentional agency inherent in human faces. One may argue against attributing mental states to facial icons or artifacts in general, asserting that there are no actual intentions or emotions behind their facial expressions. Nevertheless, information about certain emotions or intentions conveyed by variations in facial features remains accessible. For instance, one can perceive anger or aggressiveness through the facial sculptures of the Nio guardians at the entrance of certain Buddhist temples in Japan (refer to Box 1 in Supplementary materials). Obviously, saying that Nio sculptures are “frowning because they are angry” or “because they intend to engage in an aggressive act” is inaccurate. Facial icons are not rational agents endowed with attitudes or pro-attitudes (van der Hoek et al., 2007), but they may seem be behaving as such in the eye of the perceiver. Emotion is conveyed without the sender having to personally experience it. Through configurations of facial features, the facial icon would trigger the recipient to perceive an information (“convey” an information to the recipient), which is interpreted by this recipient as an emotion. The entity expressing the emotion does not require a physiological body or brain, nor does it require a will or belief underlying the conveyed information. However, the question remains regarding the conveyance of intentions. In the light of the narrative concerning Nio sculptures (refer to Box 1 in Supplementary materials), it can be argued that the anger and aggressiveness depicted in these sculptures are inferred from the thought that they protect the temples from malevolent spirits, thus the intention one can perceive in these sculptures is the one directed toward malevolent spirits attempting to enter the temple door. The latter thought stems from extended teleological explanation (Lombrozo and Carey, 2006; Roberts et al., 2020) and is dependent on the knowledge or belief related to the sociocultural context. Specifically, facial icons, being human-made artifacts, fall easily in the domain of extended teleology explanation.^6^ Furthermore, such as in animism (Descola, 2014), some facial icons may be associated with spiritual beliefs leading to the ready attribution of supernatural-agent’s intentions even without resorting to the extended aspect of teleology explanation. This perspective may serve as a means to address the indexical property of facial icons as signs. However, it relies heavily on the sociocultural context for inferring intention, making the process incomplete without the required contextual information. In contrast, the proposition advocated in this paper is independent of any specific sociocultural context and does not fall within the realm of teleological explanation. Similarly to emotion, a potential intention can be deduced from the appearance of facial icons. In this context, we refer to an intention that the sender does not plan or experience but is identifiable as an intention by the receiver through visual information. Because humans are sensitive to perceived intentions even in entities that are not endowed with intentionality (Roberts et al., 2020), such as shapes’ intentions through movements (Heider and Simmel, 1944; Tremoulet and Feldman, 2006; Pantelis et al., 2011), it is likely that intentions would be easily perceived in anthropomorphic artifacts through their facial expressions. The configuration of facial features provides valuable information much like behavioral movement does. Thus, one can discern information about an aggressive intention in the facial expression of the Nio sculptures from the combination of the facial features, which reminds the intentions of rational agents through the iconic relationship. Although the intention perceived in Nio sculptures primarily belongs to a specific sociocultural context (an intention directed toward malevolent spirits attempting to enter the temple door), we suggest that intention is “conveyed” solely through the facial expression, even when the perceiver is unaware of the spiritual narrative behind the scenes. Researchers in human-robot interaction have already exploited in their research this idea of emotion and intention that humans can detect in a computational agent (Mutlu et al., 2009; Kim and Suzuki, 2012; Schreck et al., 2019). However, in human-robot interaction situations, intentions and emotions are usually expressed through different modalities, either verbal or non-verbal such as body or facial feature movement. In this current paper, we extend the hypothesis beyond dynamic representations of emotions and intentions (such as in robot faces), and generalize our claim to encompass static expressions, given their prevalence throughout the majority of the history of figuration.

**Figure 2:**
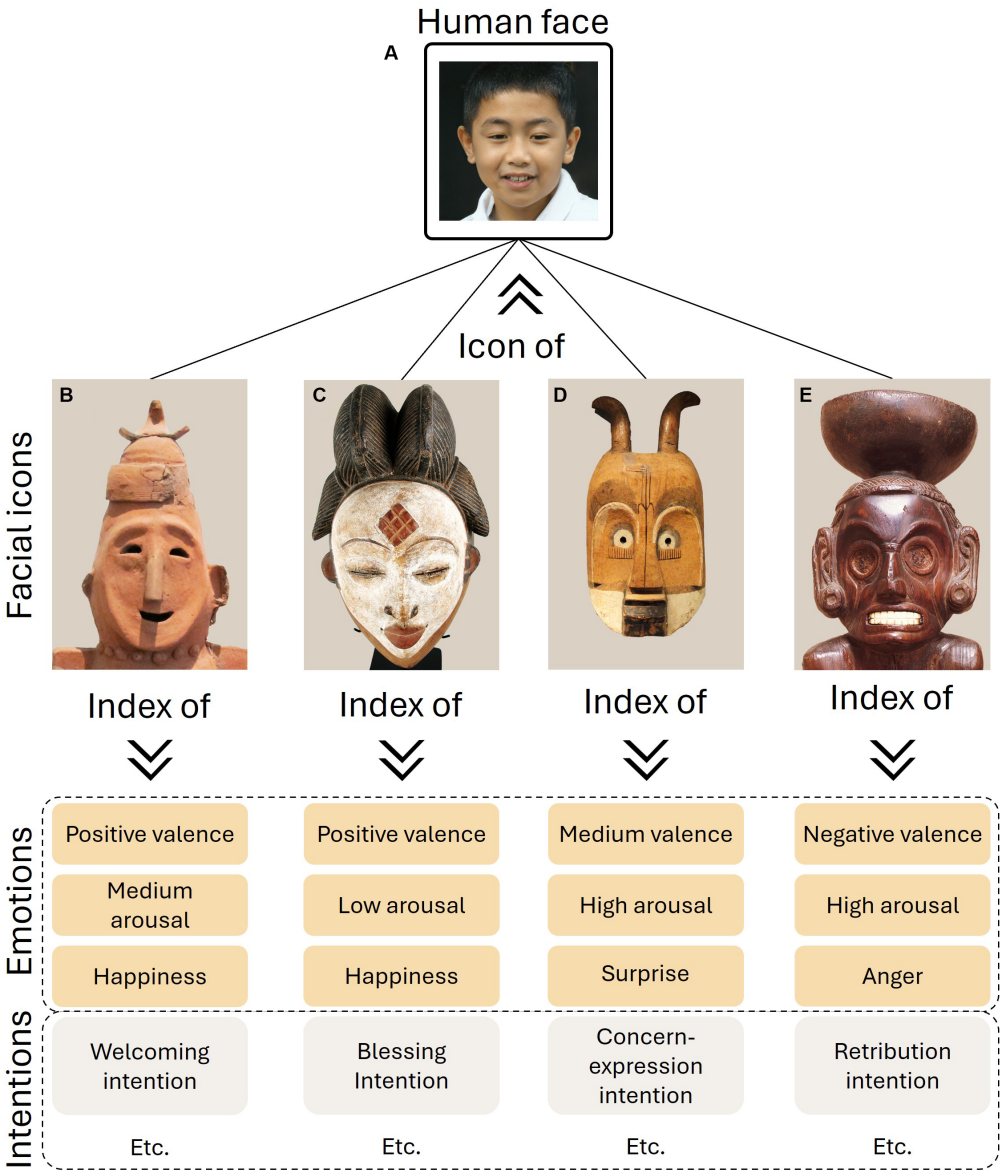
Examples of face representations from different cultures and eras and their roles in the semiotic hypothesis of facial icons. The depicted faces found in ethnographic artifacts serve as icons of the human face, establishing a resemblance with actual human facial features. Concurrently, these sculpted faces function as indexes of various mental states, as conveyed through their expressions. By communicating information regarding the causal relationship between facial configurations and associated mental states, such as emotions and intentions, these crafted faces are considered as indexes of emotion and intention. The emotions depicted in this figure, along with the corresponding facial icons, are derived from an original study scheduled for presentation at the International Society for Research on Emotion conference (Achour-Benallegue et al., 2024). The intentions represented in this figure are hypothetical and are employed for the purpose of illustrating the concept. **(A)** An image of a boy generated by StyleGAN (available at: https://commons.wikimedia.org/wiki/File:Boy_1.jpg). The person in this photo does not exist but is generated by an artificial intelligence based on an analysis of portraits. Public domain. **(B)** Figure clay, period Kofun, Haniwa. Photography by Sailko. The picture has been cropped and the background removed. Creative Commons Attribution 3.0: https://creativecommons.org/licenses/by-sa/3.0/deed.en. **(C)** Okuyi dance mask, Punu people, Gabon. Photography by Ann Porteus. The picture has been cropped and the background removed. Creative Commons Attribution 2.0: https://creativecommons.org/licenses/by/2.0/deed.en. **(D)** Mask from Cameroon (available at: https://commons.wikimedia.org/wiki/File:Mask,_Cameroon_-_Staatliches_Museum_f%C3%BCr_V%C3%B6lkerkunde_M%C3%BCnchen_-_DSC08448.JPG). Public domain. **(E)** Detail of Arawak art idol from Jamaica (available at: https://commons.wikimedia.org/wiki/File:Deity_Figure_(Zem%C3%AD)_MET_DT1258.jpg). Public domain.

In terms of information, akin to a human face, a facial icon serves as an index of emotion and intention (refer to Figure 1C). Consequently, a facial icon functions as a sign establishing a connection with emotions and intentions. In other words, when in relation to a perceiver, facial icons would exhibit an indexical behavior representing certain mental states. It is noteworthy that facial icons hold a dual significant status within the semiotic framework. These artifacts represent an iconic relationship with the appearance of the human face, hence their designation as icons. Simultaneously, the relation linking them to perceivers is characterized by an indexical connection with the mental states of human individuals (emotions and intentions). From the perspective of the relationship with the perceiver, the facial icon thus assumes the role of an index, representing specific aspects of the mental states of humans Figure 2.

## 4 Artifacts as indexes in the anthropological framework

### 4.1 Art agency

In the *art agency* theory (Gell, 1998), both artistic and ethnographic artifacts are characterized as semiotic indexes (Esquenazi, 1997; Coupaye, 2017). In this framework, the index, by indicating the cause of its existence, conveys a capacity for action, or, more precisely, a potential “agency.” Here, agency denotes a relationship of connection with a social agent. According to Gell (1998), agency is ascribed to individuals or objects perceived as instigating “events to happen” in their surroundings through acts of mind, will, or intention.^7^ In this theoretical perspective, artifacts possess attributes akin to social agents. Although it might seem unconventional to attribute agency to artifacts, this concept aligns with the notion of secondary agency advanced by Gell (1998) and embraced by other anthropologists, such as Descola (2006). The core of Gell’s theory places social agency at the forefront of the relationship with artifacts. This relationship is built on the foundation of a cognitive approach that leads to the inference of intentions implied by the artifact through a delegation of intentionality. The artifact expresses an intentionality not inherently its own but rather one delegated by a social agent. Siri Hustvedt encapsulates this idea succinctly: “Visual art exists only to be seen. It is the silent encounter between the viewer, ‘I’, and the object, ‘it’. That ‘it’, however, is the material trace of another human consciousness. The artist, who is missing from the scene, has nevertheless left us a work, an act of pure will […] The painting carries within it the residue of an ‘I’ or a ‘you’. In art, the meeting between viewer and thing implies intersubjectivity. […] The intersubjectivity inherent in looking at art means that it is a personal, not impersonal act” (Hustvedt, 2006, xix; cited in Gallese, 2017).

In the theory of art agency, the connection engendered by the index is referred to as “abduction of agency.” This denotes that, from the artifact’s indexical function, the perceiver imputes a social agency to it and formulates inferences about the intentions conveyed by the artifact. As a physical interface, the artifact functions as a conveyer of intentions, reflecting desires to act. These intentions may be actual (stemming from the human creator of the artifact) or presumed (associated with entities believed to be the source of the artifact, or those represented by the artifact).

### 4.2 The cognitive perspective of art agency

In Gell’s approach (Gell, 1998), the theory of mind hypothesis appears to be employed. The theory of mind involves the capacity to attribute mental states to oneself and others, also known as mentalization (Goldman, 2012). According to this hypothesis, all healthy human beings possess this faculty, primarily enabling them to understand and engage with others. Gell proposes that art utilizes this theory of mind for secondary, non-utilitarian purposes, such as when individuals project human mental states onto characters, whether animals or machines, in a narrative (Bloch, 1999). This cognitive perspective provides a unifying lens applicable across different cultures and eras. For instance, it allows for the exploration of relationships with robots, Flemish portraits, or Hopi kachinas alike. This is achievable because individuals in industrialized societies, 17th-century Northern Europe, or Hopi Native Americans share common cognitive mechanisms that enable them to recognize a particular agency in their artifacts. Gell’s arguments, as suggested by Bloch (1999), may draw on the work of thinkers like Dennett, leading us to consider that the theory of mind approach referenced by Gell aligns with the rationality-teleology theory (Dennett, 1987). The rationality-teleology theory, fundamentally philosophical, relies on thought experiments and extends the concept of theory of mind beyond attributing mental states solely to humans, encompassing various creatures, including animals and machines. This theory posits that a target is viewed as a rational agent that acts in accordance with its propositional attitudes. Based on its beliefs and desires, the target exhibits behavior that could be deemed rational. In this context, attributing rational behavior to a non-human target does not involve recognizing beliefs and desires in animals or machines as one does with humans. Instead, it involves attributing these mental states to the target based on the principles of the theory of mind.

## 5 Beyond art agency: experimental psychology approach

The art agency theory marked a significant milestone in the history of the anthropology of art. Its grounding in the theory of mind, despite its philosophical nature, represented a major epistemological shift in the examination and observation of social relationships with artifacts. Initiating a reflection from a cognitive psychology perspective was a logical progression from the art agency. However, when proposing that facial icons serve as indexes of emotions and possibly intentions, we do not rely on the abduction of agency as described by Gell or the internalist strategy (refer to Box 2 in Supplementary materials).^8^ We contend that attributing a psychological interiority to facial icons is not solely a consequence of their shapes portraying a body-outside prefiguring a mind-inside, but is primarily a result of perceiving this face as a human face. Research has demonstrated that various forms of facial icons, such as portraits, face-like representations, or face sketches, contribute to an increase in the negativity of the N170^9^ and elicit early brain activation in the cortical regions associated with the perception of human faces (Sagiv and Bentin, 2001; Churches et al., 2009; Hadjikhani et al., 2009; Caharel et al., 2013; Liu et al., 2016; Nihei et al., 2018). Additionally, face-likes (pareidolia faces) are linked to the rapid categorization of faces (Rekow et al., 2022). Functional imaging studies have indicated that both the encoding and drawing of cartoon faces activate face-sensitive areas in the lateral occipital cortex and the fusiform gyrus, while drawing from memory activates areas in the posterior parietal cortex and frontal regions (Miall et al., 2009). These findings support the notion that facial icons are perceived as categories of human faces, despite the varying effects of human-likeness on brain activity (Schindler et al., 2017).

Perceiving and processing facial icons as a category of human faces implies that the expressions conveyed by these artifacts could refer to an emotion, similar to the case of human facial expressions. Studies have indicated certain parallels in emotion recognition between cartoon and human faces. For instance, it has been demonstrated that, like human faces (Nummenmaa and Calvo, 2015), the recognition of happiness in cartoon faces exhibits an advantage over other emotions, with happiness expressions being discerned with greater accuracy and reduced cognitive effort (Zhang et al., 2021). Additionally, the accuracy of recognizing happiness and the perception of expression intensity of sadness in cartoons were found to be stronger compared to real faces (Zhang et al., 2021). Moreover, the communication of emotions is enhanced through facial icons. Real faces have been shown to be slower and less efficient than facial icons, such as humorous illustrations or cartoons, in conveying information, including emotion (Kendall et al., 2016). In comparison to real faces, facial icons in cartoons demonstrate higher processing intensity and speed during the early processing stage of recognizing facial expressions (Zhao et al., 2019). However, schematic faces (described as less realistic faces compared to human faces) required greater exaggeration of their features to achieve the emotional intensity of a real human face (Mäkäräinen et al., 2014). At the very least, recognizing an emotion in a facial icon could be considered a crucial step in inferring a mental state, a concept that can be translated into Gell’s terms as intentional psychology.^10^

### 5.1 Perception of emotions in facial icons

Recently, Norman & Wheeler conducted an experimental study on the perception of several masks from various cultures (Norman and Wheeler, 2020). They demonstrated that the masks evoke strong perceptions of emotion with considerable variations. Participants evaluated the masks based on the six basic emotions: happiness, sadness, anger, fear, surprise, and disgust (Ekman, 1999; Ekman and Cordaro, 2011; Izard, 2011; Levenson, 2011; Panksepp and Watt, 2011). Not only were they able to discern emotions, but they could also perceive different intensities of these emotions. Similarly, clay figures from early Japanese cultures elicited emotional perceptions in the participants (Kawabata et al., 2021). Moreover, the more the figures were perceived as happy, the more they were rated as approachable, and conversely, the more they were perceived as fearful, the less they were rated as approachable. Norman & Wheeler assert that “the ability of the masks to produce effective perceptions of emotion was due to the artists’ inclusion of facial features that reliably signal emotions in everyday life” (Norman and Wheeler, 2020, p. 1). This observation could apply equally well to clay figures from early Japanese cultures.

The signals of emotions are produced by humans across cultures and societies. In the late ‘80s, a study already explored the universality of signals,^11^ such as threat, through facial icons (Aronoff et al., 1988). This study showed that the emotional expression of threat in facial icons shares universal characteristics. American students were instructed to imagine themselves as bounty hunters from the New Guinea people, preparing for an expedition. They were then asked to draw the mask they would wear to intimidate their prey. The drawings from American students were compared with various masks from 16 different cultures, categorized as either threat-related or non-threat-related. Facial features depicting the expression of threat were identified in both the student drawings and the multicultural mask group. A more recent study further confirmed the universality of certain facial expressions (pain, anger, sadness, determination/stain, and elation) through an investigation into the perception of ancient American facial icons (Cowen and Keltner, 2020). The authors concluded (p. 4) in this study that “ancient American artists shared some of present-day Westerners’ associations between facial muscle configurations and social contexts in which they might occur.” These findings on the universality of facial expressions and emotions, spanning from contemporary Westerners to ancient and diverse cultures, support the notion that the perception of facial icons can be approached through emotion processes beyond ethnographic and cultural perspectives. It allows for the generalization of the emotion index property to a broad range of cross-cultural facial icons.

In the realm of Western art, studies have explored emotional reactions to various genres of face-representation artworks (Siri et al., 2020; Gallese, 2022), as well as reactions to face representations under different presentation conditions (Rychlowska et al., 2012). Research in the first category discovered that self-portraits elicited emotional experiences, with self-portraits being perceived as more intense in emotional expression and socially engaging compared to portraits (Siri et al., 2020; Gallese, 2022). Studies falling into the second category examined emotional responses to portraits under varying conditions of eye contact (Rychlowska et al., 2012). Artistic portraits with and without direct eye contact were presented to participants in two conditions: in the first, the represented subject’s eyes were obscured by a mask, and in the second, the represented subject’s eyes were visible. For each trial, participants answered the question “How emotional is the impact of the painting?.” The emotional impact was found to be greater when the eyes were visible compared to when the eyes were masked, particularly when eye contact was established (Rychlowska et al., 2012). This outcome suggests an embodied emotional reaction to portraits where the eyes play a crucial role.

### 5.2 Embodied emotional reactions to facial icons

We posit that facial icons, by more faithfully representing the attributes of a human being among artifacts, can be regarded as amplifiers of cognitive processes related to the theory of mind. This amplification does not rely on symbolic analogy but would be facilitated through the visual aspect of the depicted facial expressions, perceived and recognized by unconscious (cognitive) processes. The theory of mind we are invoking here is the simulation theory, characterized by “the capacity to represent and reason about others’ mental states” (Barlassina and Gordon, 2017). It involves attributing mental states by projecting oneself into the other, essentially occupying their perspective. Goldman encapsulates this concept by describing it as an attempt to generate mental states in oneself similar to those of the target (Goldman, 2012). In our application of the simulation theory, akin to Dennett’s utilization of the theory of mind (Dennett, 1987), we extend the human target “others” to the artifact target “facial icons.”

In a prior study (Achour-Benallegue et al., 2016), we observed correlations between participants’ felt emotions and their assessments of the intensity of expression in cross-cultural facial icons. The “felt emotion” variable in this study design represents a projection of the depicted emotion in facial icons, indicating the extent to which participants felt an emotion in themselves that aligns with the emotion portrayed by the facial icon. Given that the intensity of expression also reflects the intensity of the expressed emotion, this correlation suggests the presence of a simulation process toward facial icons, potentially indicating emotional contagion. Emotional contagion entails experiencing the subjective feeling state or somatosensory and motor experience of an emotion perceived in someone else (Hatfield et al., 1994; Lundqvist and Dimberg, 1995; Dimberg and Thunberg, 2012; Sato et al., 2013; Hess and Fischer, 2014; Hess, 2021). Furthermore, in another study (Achour-Benallegue et al., 2021), we explored the effect of the expression intensity of facial icons on arousal responses through subjective reports using the Self-Assessment Manikin scale. Our results demonstrated a higher self-arousal assessment among participants when exposed to intense images as opposed to neutral ones. This outcome might signify an emotional contagion suggesting a simulation of the perceived arousal affect. Emotional contagion is regarded as “basic building block of human interaction—assisting in “mindreading” (allowing people to understand and share the thoughts and feelings of others”; Hatfield et al., 2014, p. 159). When the “others” are facial icons, emotional contagion may indicate an ascription of psychological interiority to these artifacts.

Upon conducting additional analyses^12^ on our earlier study (Achour-Benallegue et al., 2016), we observed that the felt emotion (which may refer to simulation) explains 49% of the attention devoted to the facial icon, whereas the esthetic value of the facial icon explains only 4% of the attention to the image. This suggests a significant involvement of simulated emotion in the artistic relation with facial icons, where their status as indexes of emotion appears to play a prominent role.

### 5.3 Simulation through facial mimicry

**Figure 3:**
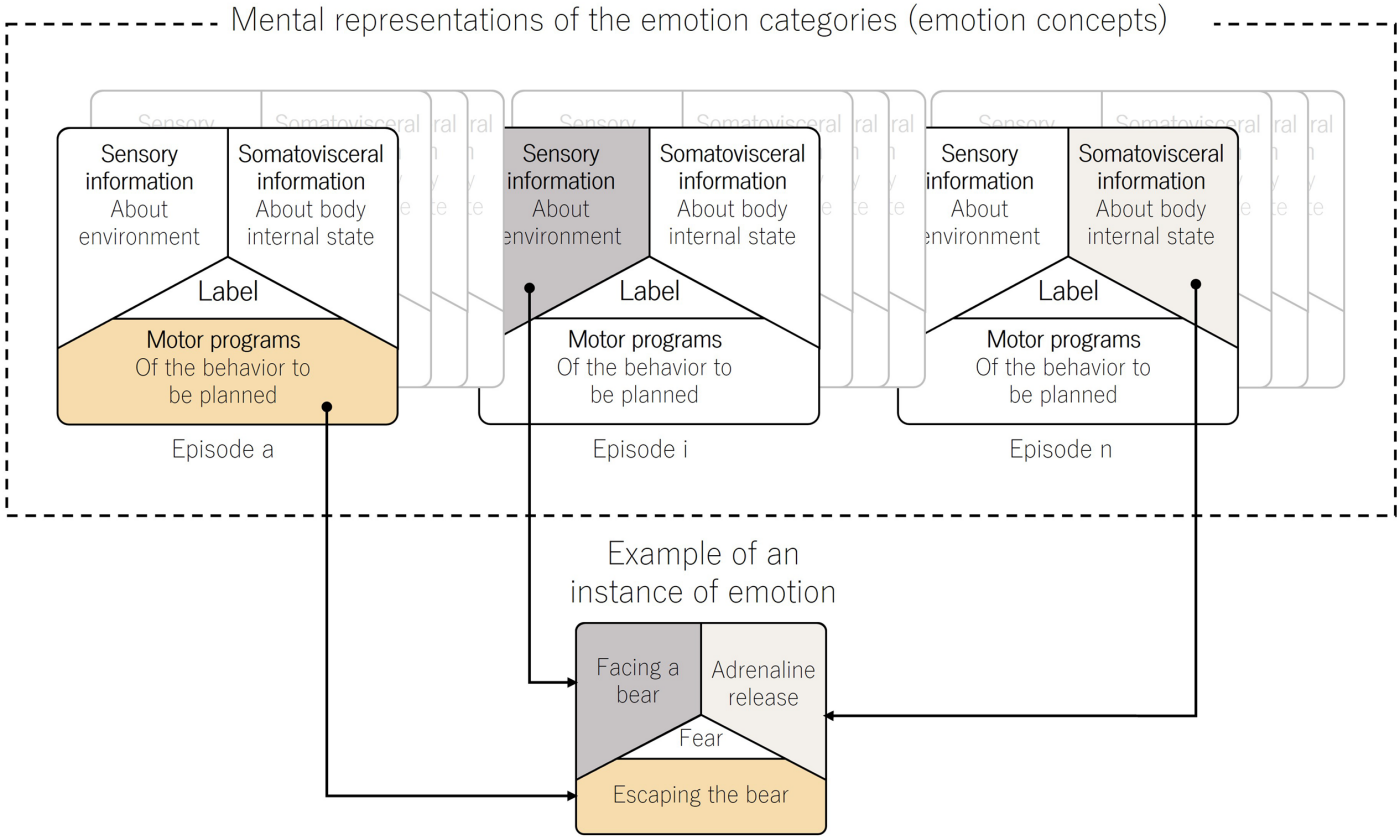
Diagram of the construction of an instance of emotion. Conceptual representation of the construction of an instance of emotion from mental representations of emotion categories, grounded in the framework of embodied emotion (Niedenthal, 2007; Barrett and Lindquist, 2008; Winkielman et al., 2008, 2015, 2018; Wood et al., 2016; Barrett, 2017). Emotions, such as fear, are experienced as *instances* accessible to consciousness. These emotion instances are depicted as distributed across mental representations of diverse emotional episodes, contributing to the emotional knowledge associated with specific categories (Mental representations of the emotion categories as emotion concepts on the upper section of the figure). The process of experiencing an emotion involves synthesizing a new instance, integrating sensory, somatovisceral, and motor modalities across various episodes of previous emotion instances. In the lower section of the figure, an example of an emotion instance (fear) is depicted. Here, ‘facing a bear’ represents the sensory modality, ‘adrenaline release’ represents the somatovisceral modality, and ‘escaping the bear’ illustrates the motor modality (the motor program of the planned behavior).

In human interaction, emotion simulation is not merely suggested by unembodied perception of emotion in facial expressions but is also triggered by the motor accomplishment of these expressions (see Figure 3; refer to Box 3 in Supplementary materials). Within the simulation theory, simulated mental states stem from sensorimotor processes^13^ that play a crucial role in facial expression processing. Motor simulation of facial expressions, that is allowed by the premotor cortex (PMC), the inferior parietal lobe (IPL), and the frontal operculum (FO), enables the recognition of others’ facial expressive movement (Gallese, 2014; Paracampo et al., 2017; Gallese, 2022). One of the essential processes that may be encompassed within sensorimotor processes is facial mimicry (Wood et al., 2016). Facial mimicry is the tendency of individuals to imitate others’ facial expressions (Hess and Fischer, 2014; Borgomaneri et al., 2020). It occurs unconsciously and spontaneously and is difficult to suppress (Dimberg et al., 2000; Bailey and Henry, 2009; Palagi et al., 2020). However, some studies showed that it may be moderated by contextual information (Murata et al., 2016; Arnold and Winkielman, 2020). Although contextual information plays a moderating role in facial mimicry, it remains a crucial factor in supporting the perception of emotional expressions. Disrupting or altering feedback from facial muscles and neural processes involved in facial mimicry impairs the processing of others’ expressions of emotion by reducing both speed and accuracy (Niedenthal et al., 2017; Borgomaneri et al., 2020). Facial mimicry plays a crucial role in shaping social and emotional interactions, immersing individuals in the simulation of another person (Beall et al., 2008; Hess and Fischer, 2013; Prochazkova and Kret, 2017). Additionally, it has the potential to result in emotional contagion (Prochazkova and Kret, 2017). For instance, when observing a smile on someone’s face, the observer may undergo the corresponding emotional experience involving the motor activation of the zygomaticus. This process involves the reconstruction of affective states associated with the sensorimotor states linked to the perception of the smile.

A recent study by Wessler and Hansen (2021) recorded facial EMG activity in response to face stimuli expressing various emotions (happy, sad, and angry), including stick figures and photographs of real humans. The study demonstrated congruent reactions of facial muscles to emotional expressions in both stick figures and photographs, emphasizing the role of mimicry even in situations with limited affiliative cues and reduced socially meaningful contexts. Stick figures not only triggered facial mimicry as much as did the photographs of real faces, but they also provided better material for recognizing emotions than photographs (Wessler and Hansen, 2021). In a previous study (Achour-Benallegue et al., 2021), we observed mimicry reactions, particularly of mouth expressions, in response to cross-cultural facial icons, suggesting that these icons might enhance a simulation process through sensorimotor reactions. In this study, mimicry reactions to the upper part of facial icons were challenging to observe, but findings on the effect of valence on muscle reactions suggested a possible tendency of mimicry reactions in the corrugator (activation of the corrugator muscle was observed in response to negative valence, while relaxation of the corrugator occurred in response to positive valence, accompanied by increased zygomaticus activity in positive valence conditions; however, in positive valence condition, the presentation of a strong-corrugator expression in facial icons impeded participants’ zygomaticus activation).

It has been suggested that in cases where the perceived facial expression is ambiguous, sensorimotor simulation might play a more significant role in emotion processing (Wood et al., 2016). The observed facial mimicry toward facial icons may indicate the involvement of sensorimotor simulation in emotion processing when dealing with facial expressions that are inherently complex and ambiguous due to their nature as freely crafted artifacts. In light of the embodied emotion theory (refer to Box 3 in Supplementary materials), the observed facial muscle reactions toward cross-cultural facial icons (Achour-Benallegue et al., 2021) and stick figures (Wessler and Hansen, 2021) provide substantial support to the simulated emotion we identified in our earlier study (Achour-Benallegue et al., 2016). These findings further endorse our hypothesis regarding facial icons serving as indexes of emotions within the framework of the simulation theory.

The neurobiological correlates of facial mimicry have been linked to the inferior frontal gyrus, a component of the classical Mirror Neuron System (MNS; Rymarczyk et al., 2018). Earlier research has posited the integration of motor and intentional aspects of action, suggesting that mirror neurons play a role in discerning an actor’s underlying motivation and intention (Rizzolatti and Sinigaglia, 2007; Rizzolatti and Fogassi, 2014, although see Ruggiero and Catmur, 2018). Moreover, the neurobiological correlates of facial mimicry extend beyond motor areas to include the neural network of mentalization, which contributes to understanding the mental states of others (Schilbach, 2016). Understanding other mental states extends beyond emotions alone, as mimicry not only relates to perceived emotions but also facilitates accurate and efficient recognition of the intention behind facial expressions (Blakemore and Decety, 2001; Wood et al., 2016). These findings align with evolutionary research, indicating that the evolution of higher cognitive abilities, such as mentalizing has been associated with the integration of perceptual and motor systems involved in face processing (Tramacere and Ferrari, 2016).

To our knowledge, experimental studies addressing the perception of intentions in facial icons are very scarce, with most of these studies originating from the human-robot interaction domain. For instance, Mutlu et al. (2009) investigated whether humanlike robots’ gaze cues and intentions can be interpreted by humans, and whether the physical design of the robot affects these inferences. Their findings revealed that gaze cues led to attributions of intentions to the robot, even when these cues were not explicitly reported, suggesting an unconscious and automatic interpretation and response. However, this effect was observed primarily in very humanlike robots and among pet owner participants. Despite the limited experimental evidence currently available for understanding the underlying intentions in facial icons, we propose that, similar to the perception of human facial expressions, the perception of facial icons could benefit from the neurobiological correlates of facial mimicry in grasping the underlying intention behind the depicted facial expressions. Drawing on studies like Blakemore and Decety (2001), motor simulation appears to be the crucial mechanism for elucidating how intentions are inferred from the shapes’ movement (Heider and Simmel, 1944; Tremoulet and Feldman, 2006; Pantelis et al., 2011). We suggest that this form of simulation, such as mimicry, also enables us to automatically infer intentions in facial icon expressions. Nevertheless, additional studies are needed to investigate and provide insights into this aspect.

## 6 Integration of the hypothesis in cultural and cognitive domains

### 6.1 Cultural domain

In anthropology and archeology materials such as artifacts remain a means to explore culture (Kumar and Ahmed, 2022). Analyzing cultures that incorporate facial icons could benefit from our hypothesis, particularly in a context where methodological recommendations are still evolving. In archeology, for example, sensorimotor simulation processing enhanced by facial icons could be included in the comparative analysis of anthropomorphic artifacts that has been conducted recently by Matsumoto (2021). Facial icons as indexes of emotions and intentions contribute to the multi-layered and complex meanings of anthropomorphic artifacts, which are regarded as a rich source of information about social structures (Rice, 2019; Matsumoto, 2021). Matsumoto (2021, p. 65) suggests that “the features of anthropomorphic figurines can provide us with clues for understanding how humans recognize other humans.” Continuing along this line of thought, we may question the role of embodied simulation of facial icons in unveiling such understandings: could the difference in sensorimotor simulation to different corpuses of facial icons reflect the differences in how the respective populations -who made the facial icons and interacted with them- perceived their peers? Previous research conducted by Matsumoto and Kawabata (2010), has already suggested that anthropomorphic artifacts (specifically Jomon clay figurines) serve as a reference point for discussions on universal human cognition. Their preliminary experimental analysis, focusing on the perception of figurine faces presented in line drawings (rather than photographs) of Japanese ancient figurines, revealed both similarities and differences between native Japanese speakers and non-native Japanese speakers. The findings suggested that our innate inclination to assess gender based on facial features is relatively robust compared to more subtle emotion reading. The authors concluded that anthropomorphic artifacts, such as facial icons including figurines, have a unique characteristic as being closely related to both social and technical cognition, encapsulating rich and complex information for their original makers and for archeologists alike. Such research, as well as the one analyzing the impressions and emotions portrayed in Japanese clay figures (Kawabata et al., 2021) represent one of the crucial steps toward integrating cognitive processes of face perception into archeological research.

In anthropology, the artifacts have traditionally been considered less significant in analysis compared to social interactions, to the extent that it has been referred to as “missing nonhumans” (Ingold, 2012). However, anthropologists such as Ingold (2012) and Woodward (2007) have emphasized the pivotal role of artifacts in construction and transmission of human culture. This has led to the emergence of approaches that consider what is commonly referred to as ‘material culture’ (Woodward, 2007; Berger, 2016). Moreover, there has been a growing recognition of the agency of material objects (Gell, 1998; Mitchell, 2005), which leads to an expansion of methods and tools for uncovering cultural and social information. In this context, our hypothesis regarding facial icons may offer an additional tool for analyzing material culture in anthropology. This can involve “setting up appropriate experimental tasks and procedures” such as suggested by Saito (2021, p. 201), that might be close to her use of children’s drawings to understand the cognitive characteristics and production processes of ancient artworks.

### 6.2 Social communication and interaction domain

Human-robot interaction is a pivotal research domain within social robotics (Sheridan, 2020), encompassing numerous studies focused on facial features in robots and their significance in social interaction such as in Mutlu et al. (2009), Carlson et al. (2019), and Stroessner and Benitez (2019), to name just a few. The field of human-robot interaction might stand to gain from the application of our hypothesis in various ways, particularly as social roboticists advocate for the integration of social psychological research into the development of social robots (Sheridan, 2020). Drawing parallels with studies like that of Dubal et al. (2011), which explores emotion perception in humans vs. low human-likeness robots, our hypothesis could shed light on psychological phenomena related to interactions with face representations commonly used in social robotics. It could be instrumental in investigations aimed at understanding reactions to robots from the perspectives of emotion and intention relying on simulation processes. Moreover, our hypothesis, along with upcoming research findings including information from morphometric features (Achour-Benallegue et al., 2024), could contribute to the design of various devices incorporating facial icons for social interactions. Robots designed for social interactions, as previously mentioned, present one potential application. For example, robot-assisted therapy seems to be a promising area in therapeutic approaches for children with autism spectrum disorders (Puglisi et al., 2022). Integrating our hypothesis into this domain could contribute to the development of next-generation devices and might potentially count among the foundational elements in designs aimed at improving the complex triadic interaction involving teachers, children, and robots.

Moreover, our hypothesis could aid in the further interpretation of findings related to emoticon perception, as seen in recent studies by Kutsuzawa et al. (2022a,b). Additionally, it could contribute to the development of new models of communication through emoticons. In summary, our hypothesis has the potential for integration across diverse domains that use facial representations to convey, communicate, inform, and enrich social meanings.

## 7 Conclusion

Various facial representations have been employed and categorized differently across various studies; nevertheless, these images collectively exhibit shared properties that justify grouping them into a unified category. The growing interest in their examination prompted us to formulate a comprehensive definition and develop a hypothesis grounded in their shared characteristic as indexes of mental states. Grounded in the theory of mind, our hypothesis aligns with the anthropological theory of art agency, which advocates a somewhat parallel perspective. While presenting our perspective, we provide arguments from embodied and simulation theory, referencing experimental studies in the cognitive scope. Our conclusion suggests that perceiving facial icons likely triggers not only the perception of emotions but also their simulation, particularly through facial mimicry. This aligns with the embodied simulation^14^ trend in art perception (the role of bodily engagement in the perception of artistic images; Freedberg and Gallese, 2007; Freedberg, 2009; Gallese, 2019; Gallese et al., 2021). Since the simulation theory is linked to understanding others’ intentions, we propose that facial icons may serve as indexes of intentions in addition to emotions.

This paper contributes to both (a) an enhanced comprehension of the cognitive experience during facial icon perception and (b) an extended interpretation of facial icons as material culture from a cognitive standpoint. On one hand, as embodied simulation of actions is potentially related to phenomenal quality (Gallese, 2017), simulating emotions when perceiving facial icons may contribute to understanding the phenomenal quality of this experience. Understanding the cognitive experience in facial icon perception can aid in designing effective tools based on facial icons for research, communication, education, or other domains. On the other hand, asserting that facial icons are effective signs for communicating emotion and intention expands the scope of material culture investigation concerning face representations. Grounding these investigations in cognitive processes enables us to incorporate artworks and artifacts from far-off locations and diverse cultures, including those about which we have scant information (De Smedt and De Cruz, 2011). For instance, aligned with hypotheses positing that the attention drawn by masks primarily relies on their activation of the fusiform face area (De Smedt and De Cruz, 2010), we propose that the comprehension of the reception of facial icons should also consider the simulation process of emotion.

### 7.1 Outstanding questions

In light of these conclusions, several compelling questions emerge, inviting further exploration and investigation into the subject, succinctly summarized as follow:

1. What morphometric features, such as eye contact, present in cross-culturally facial icons, demonstrate a propensity to facilitate emotion simulation and facial mimicry?
2. To what extent is emotion simulation from facial icons influenced by social context? If so, what types of social contexts could impact this simulation?
3. How are esthetic, ethnographic, and social experiences affected when facial mimicry is prevented in observers perceiving facial icons?
4. What role does the emotion and intention index property of facial icons play in social mediation?
5. Does the emotion and intention index property contribute to the recurring incorporation of facial icons into artistic and ethnographic traditions throughout history? Can the successful integration of facial icons into artistic and ethnographic traditions be attributed to their alignment with cognitive biases (De Smedt and De Cruz, 2010) related to face and facial expression perception?

## Data availability statement

The original contributions presented in the study are included in the article/Supplementary material, further inquiries can be directed to the corresponding author.

## Author contributions

AA-B: Conceptualization, Investigation, Methodology, Project administration, Visualization, Writing – original draft, Writing – review & editing. JP: Supervision, Validation, Writing – review & editing. GK: Supervision, Writing – review & editing. HK: Supervision, Writing – review & editing.

## Footnotes

^1^In anthropology, an artifact refers to any visual object in the world resulting from a poietic process (see Footnote 4).

^2^In this paper, anthropomorphism refers to the aspects of an object, that refer to a human or person. An anthropomorphic object is something whose appearance depicts human physical or physiognomic features.

^3^Figuration is a process of visible representation of something.

^4^Poiesis is the activity by which a person gives birth to something that did not exist before (Polkinghorne, 2012, p. 115).

^5^“Spiritual status of an artifact” refers to an artifact that appears to be endowed with a mind.

^6^The teleology explanation refers to when “something is explained with reference to its function, purpose, or goal” (Roberts et al., 2020).

^7^The use of the word “intention,” as per Gell, denotes the desire to act, to accomplish a goal.

^8^The internalist strategy is a strategy in which the artifact’s form is manipulated to create an illusion of intentionality, thereby conveying qualities associated with mental states. This approach prompts the perceiver of the artifact to infer a mental state by distinguishing the contrast between an interior (spirit) and an exterior (body).

^9^N170 is a component of the event-related potential (which is a change in the electrical potential of the cerebral cortex when the nervous system is exposed to a stimulus). The N170 is described as sensitive to faces (Eimer, 2011). Its magnitude is increased in the negative polarity direction when the stimulus is a face.

^10^Intentional psychology is used here according to Gell’s definition which refers to the possession of a mind, consciousness, etc.

^11^It is pertinent to reference an earlier but not published study conducted by Kawasaki Yuko in 1991 for a bachelor’s thesis. This study involved the rating of actual faces and ethnographic mask drawings based on the expressions of pleasure, arousal, and dominance they conveyed. The results indicated that images perceived as expressing high pleasure (high valence) led to lower self-reported pleasure, and there were no discernible differences in arousal scores between self-ratings and image ratings. Similarly, images conveying high dominance led to lower self-reported dominance, while images expressing low dominance resulted in higher self-reported dominance. Notably, masks eliciting high arousal and valence commonly featured wide-open eyes, open mouths, and visible teeth. The study suggested that masks rated low in arousal were more likely associated with burial rites and ancestor worship, whereas masks rated high in arousal were more likely used in carnivals and rituals.

^12^These further analyses conducted subsequent to the 2016 publication, were not included in the published study but are available in the Ph.D. thesis (Achour-Benallegue, 2020).

^13^Sensorimotor processes are innate processes essentially involving control of motor responses to sensory input (Bast and Feldon, 2003).

^14^Embodied simulation refers to mapping others’ facial expressions by reusing beholders’ brain regions active during the subjective experience of the same expressions (Gallese, 2014,^2022^).
