# Supplementary material for: Facial icons as indexes of emotions and intentions
Source: Front Psychol. 2024 May 14;15:1356237. doi: 10.3389/fpsyg.2024.1356237 (PMC11132266; doi:10.3389/fpsyg.2024.1356237)
Supplement: Supplementary file 1 [file Table_1.pdf]

Supplementary Material

Box 1. Nio Statue Example

The Nio statues (figure 1) exhibit a hostile expression indicative of anger. As per historical accounts (Baroni 2002), Nio statues are characterized as soldiers ready for battle, their posture and expression reflecting readiness to repel demons and other malevolent forces. Within Zen Buddhism, a prominent branch of Japanese Buddhism, the Nio sculpture holds a central significance. Specifically, Zen master Suzuki Shan (1579-1655) advised novice disciples to concentrate on the intense energy expressed by the Nio figure and to visualize it in their minds. This recommendation stemmed from his belief that they were not yet capable of engaging in the Buddha's meditation.

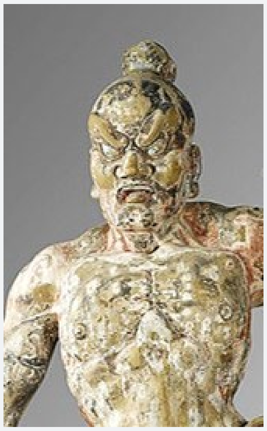

Figure 1. Statue of a Temple Guard Nio Misshaku kongo (Agyo), wood with traces of polychromy. Public domain.

Furthermore, he advocated for physically emulating the posture and facial expression of the Nio figure during meditation to generate within themselves the same level of energy. This energy, according to Suzuki Shan, should be directed towards dispelling the disturbances caused by malevolent forces during meditation. The Zen master went so far as to assert that imitating the expression and posture of the Nio statue was the sole method for beginners in Zen Buddhism to attain a meditative state (Baroni 2002).

This example provides an explicit illustration of the function of emotional expression in conveying spiritual value, a message that the Zen Buddhist community sought to convey to its adherents. Engaging in the acts of perception, imagination, or direct emulation of the emotions expressed through facial icons and body postures constitutes a fundamental practice within the teachings of Buddha's meditation. Such an approach highlights the indexical function of the facial icon, elevating its role to one that is nearly indispensable.

Reference

Baroni, H. J. (2002). *The illustrated encyclopedia of Zen Buddhism*. New York: The Rosen Publishing Group, Inc.
