# Supplementary material for: Facial icons as indexes of emotions and intentions
Source: Front Psychol. 2024 May 14;15:1356237. doi: 10.3389/fpsyg.2024.1356237 (PMC11132266; doi:10.3389/fpsyg.2024.1356237)
Supplement: Supplementary file 2 [file Table_2.pdf]

Supplementary Material

Box 2. The Internalist Strategy

The manifestation of the abduction of agency becomes evident when the index takes the form of a facial icon. Utilizing facial features to convey intentionality is not only highly effective in communicating agency but is also an economical method. Facial features attribute an "intentional psychology" [1] to the object (Gell 1998), particularly the eyes, creating an illusion of potential intentionality. Despite being aware of the artifact's non-animated nature and lack of inherent intentionality, the presence of facial features leads to the attribution of mental states to it.

According to Gell's theory, artifacts, especially anthropomorphic ones, can be imbued with a spiritual interiority (or mental state) through the internalist strategy of the mind. The transformation of an inanimate object into a quasi-person involves creating a contrast between an interior (spirit) and an exterior (body). In this strategy, the artist or craftsman intervenes in shaping the artifact to confer upon it an interiority (spirit) and provide access to this interiority. For instance, adding two dots to a disk or carving two holes in a spherical stone[2] elevates the object's status beyond that of a geometrical shape. The disk (or the sphere) transforms into an entity referencing a psychological interiority, because the two dots (or holes) - resembling eyes - suggest a *clear dividing line* (Dokic 2000) between the spirit-interior and the body-exterior. This strategy appears linked to the production phase of artifacts representing subjectivity while simultaneously serving as a means to decipher this subjectivity.

[1] Intentional psychology is used here according to Gell's definition which refers to the possession of a mind, consciousness, etc.

[2] The illustration involving holes in a sphere is drawn from Gell's analysis. In his book "Art and Agency," Gell (1998) explains that when a direct pictorial representation to confer intentional psychology (refer to the glossary) to an artifact by objectively reproducing the mind is unattainable, resorting to intermediary solutions becomes imperative. For instance, "if we cannot depict the mind, we can at least depict the possibility that there is a mind we cannot depict" (Gell 1998, p. 132). Internalist strategies constitute one such intermediary solution, manipulating the artifact's form to create an illusion of intentionality, conveying qualities of mental states.

References

Dokic, J. 2000. "Philosophie de l'esprit," in *Précis de philosophie analytique*, ed. P. Engel (Paris: PUF), 35-62.

Gell, A. (1998). *Art and agency: An anthropological theory.* Oxford: Clarendon Press.
