# Supplementary material for: Facial icons as indexes of emotions and intentions
Source: Front Psychol. 2024 May 14;15:1356237. doi: 10.3389/fpsyg.2024.1356237 (PMC11132266; doi:10.3389/fpsyg.2024.1356237)
Supplement: Supplementary file 3 [file Table_3.pdf]

Supplementary Material

Box 3. Embodied Emotion Model

Emotions are accessible to our consciousness as *instances*. This is called feeling or perceiving an emotion, such as an emotion of joy, anger, or sadness. These basic emotions (joy, anger, sadness, etc.) are categories of emotion (or classes of emotional events), each with many instances depending on contextual situations. An emotion category includes a set of instances sharing common properties (e.g., zygomaticus contraction, dopamine release), in different contexts (e.g., passing an exam, meeting a friend), but under a single label (e.g., joy). Therefore, the categories of emotion constitute emotional knowledge. In the brain, this knowledge (of which we are aware as past experiences) exists as mental representations of the emotion categories, known as emotion concepts (Winkielman et al. 2018). Ultimately, an instance of emotion is a substate of the brain resulting from an embodied cognitive process that involves emotion concepts (Barrett 2017; Niedenthal 2007).

The emotional state is associated with sensory, somatovisceral and motor modalities, which can be conceptualized as layers of past emotional episodes. Physiologically, these modalities are sensory prediction signals (perception), visceral prediction signals (affect), and motor prediction signals (action). When these modalities converge under an emotion label (e.g., joy), they create an instance of emotion (Barrett & Lindquist 2008). Given that the embodied process of emotion relies on emotion concepts formed from several previous emotional episodes, the (felt) emotion instance becomes a partial reconstruction of instances of that concept, influenced by the context (Winkielman et al., 2015; Barrett & Lindquist, 2008; Winkielman et al., 2008) (refer to Figure 2). Thus, perceiving an emotion implies reenacting in oneself the somatosensory and motor experience of the perceived emotion (Niedenthal 2007).

In the brain, emotions involve distributed and interconnected patterns of activity among a population of neurons (Barrett 2017). Activation of neurons in one modality (e.g., sensory perception) can trigger a cascade of activation in other modalities, reconstructing the instance of emotion (Wood et al. 2016). The brain's overlapping processing of perceiving and experiencing emotion leads to the natural and unconscious experience of emotion by the observer when perceiving it.

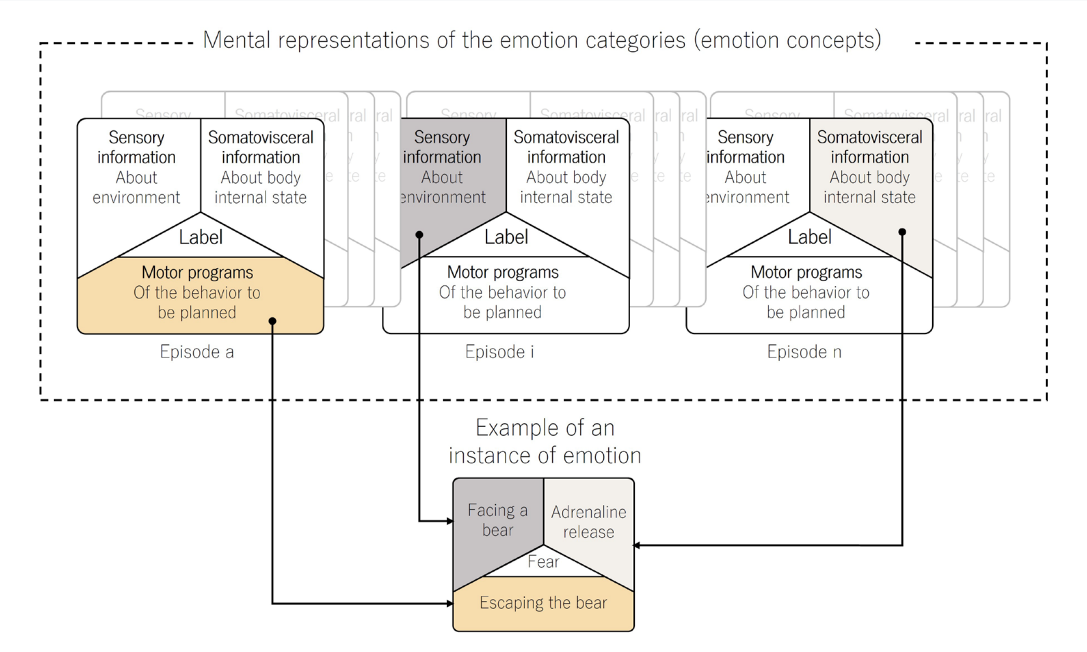

Figure 2. Diagram of the Construction of an Instance of Emotion. Conceptual representation of the construction of an instance of emotion from mental representations of emotion categories, grounded in the framework of embodied emotion. Emotions, such as fear, are experienced as *instances* accessible to consciousness. These emotion instances are depicted as distributed across mental representations of diverse emotional episodes, contributing to the emotional knowledge associated with specific categories (Mental representations of the emotion categories as emotion concepts on the upper section of the figure). The process of experiencing an emotion involves synthesizing a new instance, integrating sensory, somatovisceral, and motor modalities across various episodes of previous emotion instances. In the lower section of the figure, an example of an emotion instance (fear) is depicted. Here, 'facing a bear' represents the sensory modality, 'adrenaline release' represents the somatovisceral modality, and 'escaping the bear' illustrates the motor modality (the motor program of the planned behavior).

References

Barrett, L. F. (2017). The theory of constructed emotion: an active inference account of interoception and categorization. *Soc. Cogn. Affect. neurosci*, 12(1), 1-23. doi: 10.1093/scan/nsx060

Barrett, L. F., and Lindquist, K. A. (2008). "The embodiment of emotion," in *Embodied grounding: Social, cognitive, affective, and neuroscientific approaches*, ed. G. R. Semin and E. R. Smith (Cambridge University Press), 237–262. doi: 10.1017/CBO9780511805837.011

Niedenthal, P. M. (2007). Embodying emotion. *Science* 316, 1002–1005. doi:10.1126/science.1136930 Winkielman et al. 2018

Winkielman, P., Niedenthal, P. M., and Oberman, L. (2008). "The embodied emotional mind," in *Embodied grounding: Social, cognitive, affective, and neuroscientific approaches*, ed. G. R. Semin and E. R. Smith (Cambridge University Press), 263–288. doi: 10.1017/CBO9780511805837.012

Winkielman, P., Niedenthal, P., Wielgosz, J., Eelen, J., and Kavanagh, L. C. (2015). Embodiment of cognition and emotion. *APA handbook of personality and social psychology, Volume 1: Attitudes and social cognition*, 151-175.

Wood, A., Rychlowska, M., Korb, S., and Niedenthal, P. (2016). Fashioning the face: sensorimotor simulation contributes to facial expression recognition. *Trends Cogn. Sci.* 20, 227–240. doi: 10.1016/j.tics.2015.12.010
